# The Structures and Activities of Health Promotion in the Italian NHS

**DOI:** 10.3390/healthcare11010148

**Published:** 2023-01-03

**Authors:** Roberta Bosco, Gabriele Messina, Bruno Aiello, Giovanni Guarducci, Nicola Nante

**Affiliations:** 1 Post Graduate School of Public Health, University of Siena, 53100 Siena, Italy; 2 Department of Molecular and Developmental Medicine, University of Siena, 53100 Siena, Italy; 3 Italian Society of Health Promotion—SIPS, 80053 Castellammare di Stabia, Italy

**Keywords:** health promotion, digitalization, health project, Italian National Health Service

## Abstract

Background: In Italy, the Ministry of Health is the main decision-making entity in healthcare. The local health authorities (LHAs) are responsible for health promotion (HP) activities, based on national and regional health plans. Our aim was to investigate the structured activities of HP in Italy at national, regional, and territorial levels. Methods: From February 2020 to July 2021, we searched for online information about the structures, projects, and responsibilities at the different levels mentioned above. The sources were the official sites of the Ministry of Health, the regions, and LHAs. Results: During the “prevalence period” of 2014–2021, we found 41 active facilities dedicated to HP: 7 complex operational units and 34 simple units. The other 30 facilities also had HP activities despite the absence of dedicated units. The most discussed topic seemed to be physical activity (63%), followed by addictions (53%), nutrition (48%), and prevention (33%); in the queue appeared dental hygiene and family/parenting (both at 7%). The LHA of the City of Turin and the LHA of Salerno had the most significant number of topics. Conclusions: The results showed great heterogeneity, in the Italian context, concerning HP activities. We assume that the phenomenon depends on reduced attention to the digitalization of information. The Italian Society of Health Promotion is pursuing the goal of the construction of an organic system of HP—with its own articulations, competencies, and scientific and operational goals—at different levels, thus transcending the health care system (which is often powerless in regulatory activity) and providing the one harbinger of the most promising results in terms of cost/benefit.

## 1. Introduction

The concept of Health Promotion (HP) was born with Ottawa Charter on 21 November 1986: “Health promotion is the process of enabling people to increase control over, and to improve, their health. […] Health promotion is not only related to the health sector: it goes beyond lifestyles to focus on well-being.” [1]. This definition applies not only to health activities but also to civil life, and it requires a large investment of human and economic resources. Deciding how much to pay for health is a complex process. The Organisation for Economic Co-operation and Development (OECD) states that, in the countries of the European Union, the per capita spending on health in 2019 was about 8.3% of the GDP—from over 11% in Germany to less than 6% for Luxemburg and Romania [2]. In Italy, the Ministry of Health (MH) is the main actor in health; together with other ministries (economic, education, environment, labor, etc.), it spends €2473.00 per capita [2] to meet the needs of the population, which has been deduced from epidemiological studies and discussions with the Italian regions. The MH drafts the National Health Plan (NHP), which at the state level, is the main planning tool and fundamental principle in the field of “health protection” [3]. However, health care responsibilities, under health federalism, switched to the regions after a series of amendments to the Italian Constitution [4,5] and the new identification of the essential levels of care, which were divided into three main areas: collective prevention and public health, district care, and hospital care. The first falls of chronic disease surveillance and prevention included the promotion of healthy lifestyles and organized screening programs, nutrition surveillance, and prevention [6]. Currently, the 2006–2008 NHP [3], which covers both prevention and HP topics, is valid. On 6 August 2020, to better define what is related to prevention and health promotion, the State–Region Permanent Conference approved the National Prevention Plan (NPP) 2020–2025, which is key in planning prevention and health promotion interventions to be implemented in the territory [7]. The new NPP has both a SARS-CoV-2 pandemic part and an HP part as central points of health policy “to prevent the disease and strengthen practitioners’ skills and awareness” [7]. Regions are required to adapt their Regional Prevention Plans (RPPs) to the national one by a set date (in this case, 31 December 2020). The activities indicated by the RPPs are assigned to the organizing facilities (simple or complex units), that report to the LHAs. Healthcare workers assigned to these units are responsible for the prevention, health promotion, and education of individuals, families, and the community [8]. The number and variety of specialists working there, the level of technology achieved, and above all, the ability to carry out actions in support of the authorities, to ensure the proper implementation of the RPP, to coordinate the various professional processes, and to mobilize economic resources, determines the degree of complexity of the different units. The simple unit, while having autonomy in the management of human, technological, and financial resources, either reports to the complex units or to a department (the macro structures that includes all the units working on similar issues (e.g., the Department of Prevention), or they report directly to the Health Directorate [9]. According to Legislative Decree 502/1992 and the subsequent Legislative Decree 299/1999, the Department of Prevention is divided into different areas that deal with different aspects of public health: the facilities of “Hygiene and Public Health” and “Food and Nutrition Hygiene” may deal with, among their projects, issues of HP. The latter, among his or her roles, has precisely that of dealing with the promotion of healthy eating in public facilities, such as schools, hospitals, and accommodation facilities [10,11]. There are also organized projects with a head for which there is not necessarily a dedicated facility, which may have a defined period, respond to a request from the Directorate, lead collaborations between several intra- or extra-local facilities or authorities, or others. Finally, there are extemporaneous initiatives of various facilities, often undertaken in collaboration with scientific societies or voluntary or other associations, which are part of the panorama but cannot all be reported with the hope of completeness. Given the heterogeneous nature of the organization of HP in Italy, we aimed, under the auspices of the Italian Society of Health Promotion, to identify structural (and therefore recordable) activities—by analyzing the organization and management of HP activities in Italy, at the national, regional, and territorial levels, from the central office to the operational units (OUs) of LHAs—of dedicated and formally established projects.

## 2. Materials and Methods

The research was conducted online from February 2020 to July 2021. The reference sources were the official websites of the Ministry of Health, the regions, and the LHAs. Unfortunately, not all regions update their RPPs or their websites with changes in organizational charts and/or new projects in a timely manner. The investigation was about:

1. Who is responsible for HP at a national level?
2. Which central regional structures deal with HP and what issues do they develop?
3. How many LHAs have facilities dedicated to HP?
4. How many and what projects do the LHAs have and what topics do they develop?
5. What are the independent institutions/associations that promote health?

Data were collected, organized, and analyzed using Microsoft Excel (ver. 16) software. To answer point 1, organizational charts were searched for on the national government’s official website.

To answer point 2, the research was performed on regional organizational charts for directorates or departments dealing with HP, going into detail down to the offices/OUs, whose main purpose is HP. To achieve this, we used the same methods applied in step one. We analyzed the RPPs (2014–2018) of the different regions to estimate the importance, at least on paper, that they devoted to HP, based on the NHP indications (we searched for the word “promotion” within each document, either alone or associated with different concepts: health, healthy lifestyles, healthy behaviors, healthy food consumption, etc.). To collect the HP initiatives of the above-mentioned structures, the search was conducted in the thematic sections of the regional websites by searching for the terms “health” or “prevention.”

To answer point 3, the words “organizational chart” or “health promotion” were entered in the search box of each site. From the results obtained, we proceeded to obtain either the PDF documents showing the actual organization of the offices or the direct link to the HP section of the site. The database resulting from this first phase was organized according to:

- Name of the region;
- Name of the LHA;
- HP status (with 2 indicating the LHAs that had a simple or complex unit dedicated to HP, 1 indicating the LHAs that dealt with the topic without having a dedicated facility, and 0 indicating that no traces of the topic were discerned on the site);
- Type of unit (simple or complex);
- To which complex unit the simple unit reported to;
- To which department/directorate the simple or complex units reported to.

To answer point 4, the results of the inclusion of the words “health promotion” and/or focusing on the sections of dedicated facilities, where present, were explored. All projects or initiatives promoted by the LHAs on their websites were collected, paying attention to the year of reference, as some were named but not active, and to the degree of depth and accuracy of their description. In a second step, the active projects were classified into macro-areas that could indicate the main themes of the HP, as follows:

- Physical activity
- Nutrition
- Sexual health
- Addiction
- Safety in the home
- Safety on the street
- Mental health
- New technologies
- Women’s health
- Workplaces
- Discrimination
- Dental health
- Maternity/breastfeeding
- Prevention (screening programs)
- Family/parenting
- Environment
- Other (blood, organ and tissue donation, migrant health, life skills, domestic violence, first aid, COVID-19).

In addition, to obtain feedback on the sensitivity/reliability of the method applied, preliminary results were sent to representatives of four regional entities (Lombardy for the north, Emilia Romagna for the center, Campania for the south, and Sardinia for the islands), which were drawn on a voluntary basis by the Board of Directors of the Italian Society of Health Promotion.

To answer point 5, the source used was the Italia nonprofit website (italianonprofit.it) (accessed on 12 July 2021). It aims to survey all associations involved in health. In addition, a Google search was also conducted by typing “health promotion in Italy.”

## 3. Results

The results showed what appears (or rather, appeared) during the studied period and not necessarily what is currently active. It is a “periodic prevalence” of structures, projects, and activities in place from 2014 to 2021.

### 3.1. Answer to Point 1

At the national level, the analysis of government sites showed that it was mainly the MH that dealt with HP, as it was entrusted to the Directorate General of Health Prevention, in particular, Office 8 “Health Promotion and Prevention and Control of Chronic Degenerative Diseases.” In addition, other relevant HP activities were carried out by the Department of Anti-Drug Policies and the Office for Policies in Favor of Persons.

Office 8’s initiatives include:

- National Prevention Plan;
- Health Gain (tobacco, alcohol, nutrition, and physical activity);
- Tumors (national network of rare cancers, screening);
- Global Alliance Against Chronic Respiratory Diseases—GARD Italy.

The Italian National Institute of Health (INIH), the main technical scientific body of the National Health Service (NHS), deals with HP through the National Center for Disease Prevention and Health Promotion and the Department of Cardiovascular, Endocrine-metabolic and Aging Diseases. The first one was responsible for the national coordination of disease prevention and HP initiatives, researching and testing effective HP methods, building integrated multidisciplinary approaches, and promoting sustainable evidence-based interventions. The second one had strategic activities in the areas of alcohol, nutrition, breastfeeding, physical activity, life and working contexts, accidents and injuries, obesity, and lifestyles.

### 3.2. Answer to Point 2

**Figure 1:**
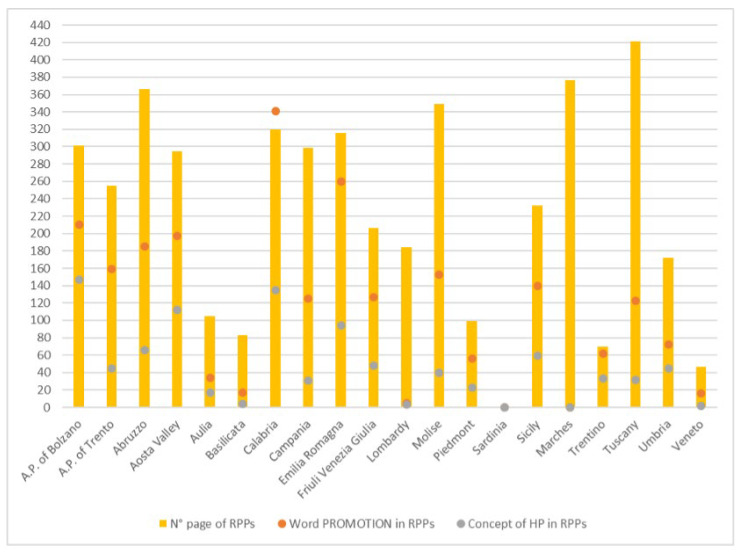
Comparison of document consistency and health promotion expression, in terms of word count and concept.

Figure 1 shows the results of the analyzed RPPs.

There is no downloadable RPP from the Latium website, so the document was downloaded from epicentro.it (accessed on 15 July 2021).

For the Marches region and Sardinia, word repetition could not be quantified because the format of the online document did not allow for this search. Sardinia’s PRP was presented in several sections, with documents related to each program, so a calculation of pages in total was not possible.

Table 1 shows how many regional departments had a unit dedicated to HP (12 out of 21 = 57%) and how many topics, listed earlier in Section 4 of “Materials and Methods,” were named on their websites. The environment was not considered among them, as there has always been a dedicated section on the regional sites. Physical activity, addiction, and prevention were among the most covered topics, with 15, 12, and 12 regions out of 21, respectively, having these topics. In the category “other”, protection of prisoners’ health appeared frequently, while the theme of new technologies remained at 0. For the Calabria region, although a structure dedicated to prevention and public health was visible in the organizational chart, it was not possible to assign an HP theme because nothing appeared on the site. For Piedmont, Campania, Valle d’Aosta, and Sardinia, it was not possible to understand to whom the HP was assigned; the last two, moreover, had a site that was not very up to date.

### 3.3. Answer to Point 3

**Figure 2:**
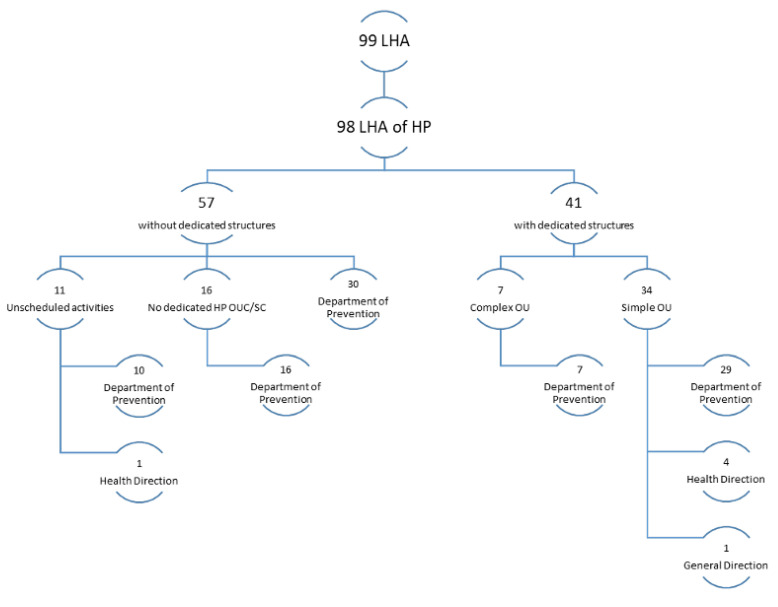
Diagram of hierarchies in Italian LHAs for health promotion.

In Italy, there were 41 facilities dedicated to HP, of which 7 were complex operational units (COUs), and all of them reported to a department (of prevention, hygiene and health prevention, or public health). Of the simple units (SUs), most (29) reported to one of the above departments, 4 to the Health Directorate, and 1 to the General Directorate. The LHAs that did not have any dedicated OUs carried out HP activities with projects and initiatives coordinated directly by the facilities dealing with related issues (public hygiene and health, food hygiene and nutrition, etc.) or directly by the prevention department, as was the case for 30 out of 57 cases (Figure 2).

It is specified in Figure 2 that the departments under the heading “Departments of Prevention” also included those that took slightly different names, according to the regions, while maintaining prevention as their main mission—albeit with some different targets according to the type of prevention work (e.g., hygiene and health prevention, public health, veterinary prevention department, etc.).

**Figure 3:**
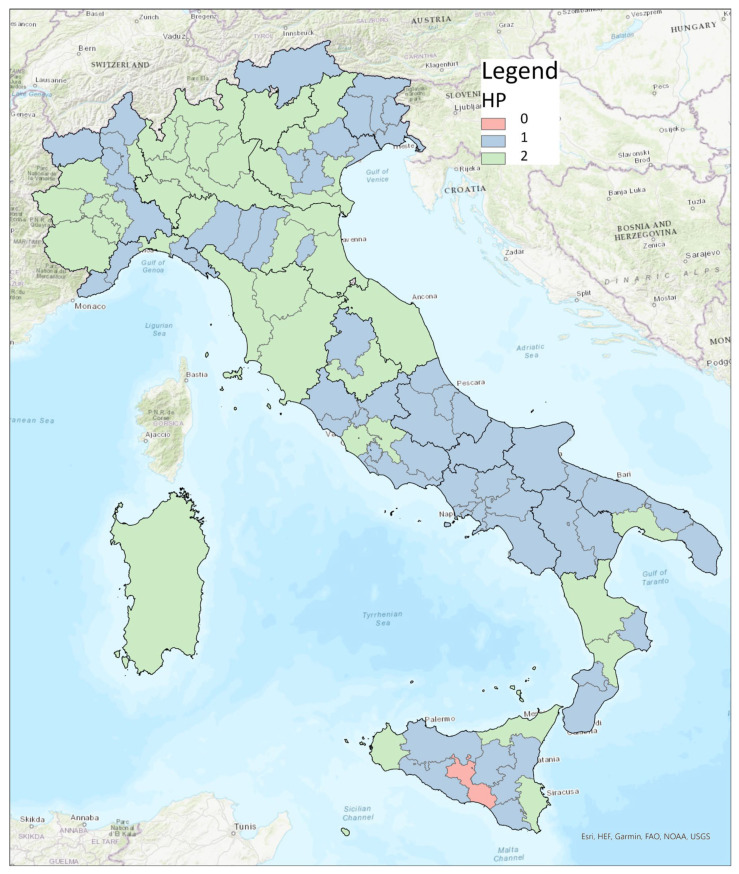
Distribution of LHAs involved in health promotion in Italy.

Figure 3 shows that the northern and central regions of Italy had a higher percentage of facilities dedicated to HP, while although there were activities on the topic in the southern region, there were no specific facilities for these projects.

### 3.4. Answer to Point 4

The HP issues addressed by the LHAs’ projects in Italy are distributed by the order of frequency in Table 2 below.

**Figure 4:**
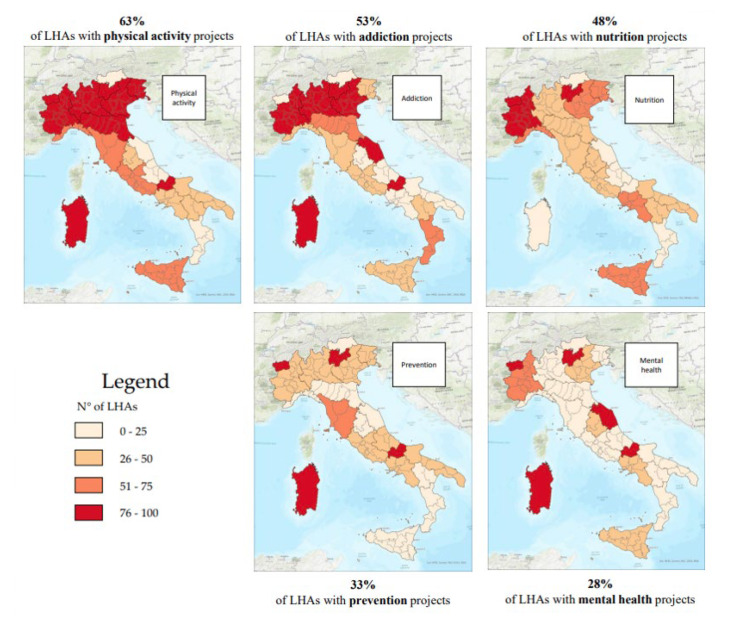
Distribution on the Italian territory, divided by the regions whose LHAs have projects dedicated to the topics indicated.

The most discussed topic, regardless of whether they had a dedicated facility, appeared to be physical activity (63% of cases) followed by addictions (smoking, alcohol, and drugs), while dental hygiene and family/parenting appeared to be less addressed (in only 7 out of 99 cases). The LHA for the City of Turin (which had no dedicated facility) and the LHA of Salerno had the highest number of topics addressed (12 out of 17, if the category “other” was included), followed by 3 others in Piedmont, 2 of which had no dedicated facility, and the Provincial Health Authority (PHA) of Messina. In five cases, no actual projects were found, and only dissemination materials were present. It was not possible to fully evaluate the offerings of eight of the LHAs, either, due to the lack of explicit references to projects or due to the last site updates dating back at least three years (2018). Among the latter, the LHA of Umbria 2 totaled zero projects under its charge, although it had a dedicated Departmental Simple Unit (site updated in 2017). Along with it, the LHA of Montagna (Lombardy), LHA 5 of Polesana (Veneto), the LHA of Piacenza, the LHA of Catanzaro, and the LHA of Siracusa had dedicated facilities but had projects for less than two HP themes. In detail, we noted that Abruzzo, Calabria, Marches, and the A.P. of Bolzano had zero facilities promoting physical activity, while Apulia and Campania had less than 50%. There were only five regions that had 100% of LHAs dealing with this issue. However, apart from Friuli Venezia Giulia, which had three LHAs, the other regions (Molise, A.P. of Trento, Sardinia, and Aosta Valley) had only one LHA. After integration, Emilia-Romagna rose to eight LHAs promoting physical activity, thus achieving 100%. Figure 4 shows the distribution, at the national level, of the top five topics most frequently dealt with by the various LHAs.

The main discrepancies found after receiving the additions from the sample regions concerned the activities of the LHAs of Piacenza, Parma, and Reggio Emilia, which reported having a larger number of projects. It should be noted that additions may have been made after the survey period.

### 3.5. Answer to Point 5

From the analysis of a site known as “Italia nonprofit”, which went through the trouble of collecting information on various sporadic HP initiatives in Italy, out of the 110 associations found, only 1 (Aghape) had HP among its objectives—albeit in a distinctly “holistic” sense. All other associations dealt specifically with precise issues (e.g., HP for those suffering from drug addiction, for alcohol abusers, for cancer patients, for migrants, etc.). From the Google search, however, we found associations that are already structured and have been known to deal with the issue for some time:

- CIPES—Center of Initiative for Health Promotion and Health Education (Turin, 1990)
- ISES—European Institute for Economic Development (Alexandria, 2010)
- Health Prevention Education (Turin, 2020)
- Dors—Regional Documentation Center for Health Promotion (Grugliasco-TO, 1998)
- CeSPES—Experimental Center for Health Promotion and Health Education (Perugia, 1954)
- CREPS—Research Education Prevention and Health Promotion (Siena, 1999).

## 4. Discussion

Measuring health is not easy, given its definition [12]. To invest in HP activities, however, it is necessary to analyze health needs through epidemiological indicators, and this is what the NHS does [13]. The OECD, through its “Health at a Glance” section, gives us a quick overview of the health situation in Europe, focusing on issues of importance to HP, such as air pollution, obesity, smoking, alcohol, and inequalities [1]. It emphasizes how important everyone’s participation (individual and collective) is to achieve the goals of proper HP activities [14]. The concept of “Health in All Policies”, advocated for by WHO [15]—which should be a key tool for recognizing the importance of public health policies [16]—is far from finding substantial application. In fact, to date, in Italy, it is the MH that deals with health, and this research has been focused on it. The MH dedicates a special office to HP that devotes itself to the fight against smoking, alcohol, and respiratory diseases; it is also involved in prevention, especially in cancer [17]. In addition, there is the Italian National Institute of Health (INIH), which has strategic activities dedicated to different thematic areas typical of HP [18]. The 2014–2018 PRPs are all based on the PNP, with some adjustments based on the specific needs of each region. Yet, if we analyze them, as shown in Figure 1, we note how the concept of HP is most expressed by the A.P. of Bolzano and by Calabria; the latter also holds the record for the number of repetitions of the word “promotion,” which is often associated with concepts of HP in the documents. It should also be said that the types of HP activities undertaken were also dissected in other types of documents, which were not always on the sites or readily available. Analyzing Figure 3, however, we can see that the apparent attention given in the RPPs to HP did not reflect the real commitment that the individual regions had invested in the creation of specific projects. First, we can see that only 57% of the regions had an HP unit and that Calabria itself had no HP project tracked on the site. The percentage dropped to 41% when we analyzed individual LHAs, with the one in Caltanissetta having had no HP reference on its site. Out of 21 regions, 11 (52%) fell below the 3rd quartile in terms of the presence of dedicated HP facilities in their LHAs, 6 of which did not even have one. From analyzing how HP was organized at a structural level in the Italian LHAs (Figure 2), we saw that among those that were not present in the organizational chart but were carrying out HP activities, only in one case were such activities assigned directly to the Health Directorate; four simple OUs reported directly to it, and one reported to the General Directorate. Among the 57 LHAs without a dedicated OU and the 41 with a dedicated OU, there were 92 LHAs entrusted the HP to the Prevention Department. This is an agreeable choice, considering that health promotion can be included in primary prevention, even though—given the multitude of issues it deals with and the countless activities and projects that could be carried out to reach the population—it would be appropriate to dedicate an entire OU to proper resource management. Although it is, indeed, “primary”, it is not taken for granted that the same range of initiatives for the entire catchment area can be found in the same region; the percentage distribution of HP topics addressed by the LHAs started from a maximum national coverage value of 63% and decreased to a minimum of 7%, although the RPP was valid for every LHA. It is also true that each area has special needs. If we take, for example, the macro area of physical activity, which could be considered an issue that should be indiscriminately promoted everywhere, and which represents an excellent investment for Italy, as shown by Goryanik Y. Et al. [19], almost no region presents complete coverage (if we exclude Friuli-Venezia-Giulia and those with only one LHA). Even if we consider the apparently more virtuous regions, in terms of the topics covered (Figure 3), such as Piedmont, Lombardy, Veneto, and Sicily, we find an average coverage of 79%; this means that some of their areas are not involved in projects promoting physical activity. The percentages decreased if we analyzed the other areas. During the period of our research, the WHO Health Promotion Working Group [20] published manuals and guidelines on health promotion in schools, a topic that we saw appearing frequently in our survey. Many projects promoting physical activity, healthy eating, which has always been of great importance in HP [21], and the correct use of new technologies, take place in schools thanks to the alliance between the “Gaining Health” initiative and educational institutions [20]; however, the WHO also focused on urban health [22], which never appeared in the analyzed sites. Since, during the pandemic period, the Internet was almost the only channel of communication with citizens, it is thought-provoking to find the sites of healthcare providers out of date. The NRRP (National Recovery and Resilience Plan) [23] has “digitization and innovation” as one of its main intervention strategies, which have increasingly been key in the communication and sharing of current news and knowledge. Methods and approaches to HP are also constantly evolving, so training future professionals involved in each field is critical. As mentioned by A. Mereu et al., it is important that there are basic skills present, upon which more specific skills can be built; the former to strengthen the workforce, the latter to improve the efficiency and effectiveness of activities in HP [24].

## 5. Limitations

The research method used in our survey was inevitably affected by the degree of updating of the Health Boards’ websites and, therefore, has limitations that could have caused us to underestimate or, less likely, overestimate, the current level of HP in the NHS. There is also the possibility that there are dedicated sites that are not reported on the web pages of LHAs or on their official social networks, which do not appear as possible “contacts”. This does not help the orientation of the common citizen. The research was stopped in July 2021, both because, at that time, the data collection seemed adequate for all regions and because, due to the pandemic, normal activities were both live and online.

## 6. Conclusions

Nowadays, the Internet represents the main communication channel for the population. The importance of strengthening life skills for profitable use of this tool is crucial, and it is also emphasized by Fioretti G. et al., especially, to rethink effective HP activities [25]. The uneven coverage that appeared from our research claims an unequal distribution of health services in our country, at least as far as the topic under investigation is concerned. Our research mapped HP activities and structures within the Italian territory, dividing them by individual regions and topics. This showed heterogeneity in the distribution, reporting an uneven distribution of health services in our country, at least with respect to the topic investigated and with all the previously listed limitations of the methodology used. For future research purposes, it would be interesting to interview the citizens to verify how effectively the various LHAs are able to reach their catchment areas with HP activities. If the problem were only in the mode of communication [26], it would be a matter of standardizing the quality and quantity of information to increase adhesion to the various awareness campaigns, which already must overcome prejudices and psychological factors that make promotion and prevention interventions complex [27]. Far more serious would be if the mismatch were substantial and not just apparent, with the substantial betrayal of two fundamental principles of our NHS: equity and equality [28]. By investing in HP activities, we are convinced that the cost–benefit balance is even more favorable than that of primary prevention activities [29]. We hope that our work can be a stimulus to further research—with more time and energy, even before receiving additional economic resources—which would require careful analysis before being properly distributed [30]. Our NHS, let’s face it, produces quantifiable and objective health outcomes. Now it appears to be mainly responsible for interventions and investments—not only in prevention, diagnosis, and the treatment of diseases—but also in HP, which according to the World Health Organization, should be present in all policies. The Italian Society of Health Promotion continues to pursue the goal of advocating for the construction of an organic system of HP—with its own articulations, competencies, and scientific and operational goals—at different levels, thus transcending the health care system (although it integrates some functions, especially in the field of prevention), which is often powerless in regulatory activity. This presents the most promising results in terms of costs/benefits.

## Figures and Tables

**Table 1:** Central structures specifically dedicated to HP in the departments of the Italian regions.

REGION	HP Unit	N° of Topics	Macro-Structures
A.P. of Bolzano	Yes	4	Health Department
A.P. of Trento	No	4	Health and Social Policy Department
Abruzzo	Yes	8	Health Department
Aosta Valley	No	5	Health and Welfare Department
Apulia	Yes	7	Department of HP and Animal Welfare
Basilicata	No	5	Personal Policies Department
Calabria	Yes	0	Health Protection, Social and Health Services Department
Campania	No	3	General Directorate for Health ProtectionGeneral Directorate for Social-Health Policies
Emilia-Romagna	Yes	8	General Directorate for Welfare
Friuli Venezia Giulia	Yes	6	Central Directorate of Health, Social Policies, and Disability
Latium	Yes	5	Regional Directorate of Health and Socio-Sanitary Integration
Liguria	No	6	Department of Health and Social Services
Lombardy	Yes	6	General Directorate for Welfare
Molise	No	2	General Directorate for Health
Piedmont	No	7	General Directorate for Health and Welfare
Sardinia	No	2	Health Department—SardegnaSalute
Sicily	Yes	5	Health Activities and Epidemiological Observatory Department
Marches	Yes	4	Regional Health Agency
Tuscany	Yes	5	Directorate of Welfare and Social Cohesion
Umbria	No	5	Directorate of Regional Health and Welfare
Veneto	Yes	3	Directorate of Prevention, Veterinary Food Safety

**Table 2:** Percentage of LHAs in Italy having projects dedicated to a specific topic.

Topic	% of LHAs that Have Activities Dedicated to the Topic
Physical activity	63%
Addiction	53%
Nutrition	48%
Prevention	33%
Mental health	28%
Other	27%
Workplaces	24%
Sexual health	23%
Maternity/breastfeeding	15%
Safety in the home	15%
Safety on the street	15%
New technologies	14%
Environment	13%
Women’s health	12%
Discrimination	8%
Family/parenting	7%
Dental health	7%

## Data Availability

Not applicable.
